# Hypocomplementemic Atypical IgA Vasculitis: A Case Report

**DOI:** 10.3389/fped.2022.886371

**Published:** 2022-06-09

**Authors:** Melvin Chan, Melisha Gayle Hanna, Nicholas Willard, Amy Treece, Bradley Patton Dixon

**Affiliations:** ^1^Department of Pediatric Nephrology, Children's Hospital Colorado, Aurora, CO, United States; ^2^Department of Pathology, School of Medicine, University of Colorado, Aurora, CO, United States

**Keywords:** IgA vasculitis, case report, hypocomplementemia, HSP, membranoproliferative glomerulonephritis

## Abstract

IgA vasculitis (IgAV, also known as Henoch-Schönlein purpura or HSP) is a vasculitis of small vessels involving multiple organs, particularly of the joints, gastrointestinal tract, skin, and kidneys. Growing laboratory evidence has shown that complement plays a key role in the pathogenesis of IgAV, although direct evidence of this association in patients is lacking. We report a child with IgAV associated with clinical features of hypertension, nephrotic range proteinuria, acute kidney injury, and low serum C3, with histopathologic findings on renal biopsy of membranoproliferative glomerulonephritis with C3 and IgA co-dominance, and extensive complement derangements. This case report suggests that complement modifies the pathogenesis of IgAV, and further investigation into complement-targeted therapy in cases of refractory IgAV may be beneficial.

## Introduction

IgA vasculitis (IgAV, also known as Henoch-Schonlein purpura or HSP) is a vasculitis of small vessels involving multiple organs, particularly of the joints, gastrointestinal tract, skin, and kidneys. The diagnostic criteria for IgAV is the presence of palpable purpura with at least one of the following clinical features: abdominal pain, arthralgia/arthritis, renal involvement with glomerulonephritis; or skin biopsy of the purpuric lesion demonstrating leukocytoclastic vasculitis with predominant deposition of IgA. Renal involvement (IgA vasculitis nephritis, or IgAVN) occurs in 20 to 55% of patients within 3 months after the development of purpura, with 85% of nephritis cases presenting within the first month (1, 2). Biopsy of IgAVN typically reveals IgA immune complexes with mild mesangial proliferation and expansion of the mesangial matrix, and may include segmental necrotizing inflammatory lesions or crescents (1). Such pathological findings are similar to IgA nephropathy, with the exception that immune complex deposition in capillary loops is more frequently observed in IgAVN (3). IgAVN may also have a membranoproliferative pattern, but it remains a rare phenomenon (4, 5).

IgA immune complexes are hypothesized to play a central role in the pathophysiology of IgAV. These immune complexes are formed following an antigenic exposure and deposit in small blood vessels including the glomerular capillaries and mesangium, resulting in recruitment of inflammatory cells. Aberrant glycosylation of IgA in the hinge region has been demonstrated in both IgAVN as well as IgA nephropathy (6, 7). The galactose-deficient IgA-containing immune complexes likely activate the complement system through the lectin pathway, as deposition of complement protein and fragments such as C4d, mannose binding lectin (MBL), L-ficolin, and MBL-associated serine proteases (MASPs) in the glomeruli are associated with higher grade proteinuria and hematuria as well as more severe histological lesions in IgAVN (8). More recent work has revealed that collectin-11 has been found to bind to IgA-antibody complexes and activates complement through the lectin pathway, further supporting the effector role of complement in the pathogenesis of IgAVN (9). Despite the clear role of complement activation at the tissue level in this pathogenesis, serum levels of complement proteins tend to be normal in IgAV (10). In a series of patients published by Lin et al. hypocomplementemia (low C3, low C4, or low C3 and C4) was noted in 15% of patients with IgAV, indicating activation of the classical or lectin pathways (11). However, evidence of complement activation did not confer an increased incidence of IgAVN, or more severe renal involvement (10, 11).

We report a child with IgAV associated with clinical features of hypertension, nephrotic range proteinuria, acute kidney injury, low serum C3, and normal serum C4, with histopathologic finding on renal biopsy of membranoproliferative glomerulonephritis with C3/IgA co-dominance by immunofluorescence, and extensive characterization of the complement system revealing activation of the alternative pathway, without clear activation of the terminal pathway. To our knowledge, there are few case reports of similar presentation in the literature (4, 12), none of which have been subjected to an in-depth interrogation of the complement system.

## Case Presentation

A 5-year-old previously healthy Native American boy presented to a local emergency room with pruritic palpable rash in the lower and upper extremities, associated with abdominal pain and hand swelling. His family medical history was negative for autoimmune disease or renal disease. Blood pressure was 90/60 (normal systolic range 80–100; normal diastolic range 40–60). Initial examination revealed a purpuric rash more prominent on the lower extremities than the upper extremities with mild tenderness to palpation of the abdomen. Laboratory values revealed a c-reactive protein (CRP) 30 mg/L, prothrombin time 11.4 s, international normalized ratio (INR) 1.1, partial thromboplastin time (PTT) 29 s, white blood cell count (WBC) 10,200/μL (neutrophils 53%, lymphocytes 35%, monocytes 8%, immature granulocytes 1%, and eosinophils 3%), hemoglobin 12.3 g/dL, platelet count 261,000/μL, blood urea nitrogen (BUN) 10 mg/dL, creatinine 0.29 mg/dL, albumin 2.9 g/dL, and normal liver enzymes. A skin biopsy was not undertaken, but based on the features of his clinical presentation, he was diagnosed with IgAV. The patient was discharged home and instructed to follow-up with his pediatrician.

At a follow-up visit with his pediatrician 2 weeks later, his symptoms had resolved with the exception of his abdominal pain. His blood pressure was then noted to be 130/80 with mild edema of his extremities on examination. The purpuric rash had resolved. Laboratory investigation on Day 18 of illness revealed BUN 62 mg/dL, creatinine 0.57 mg/dL, albumin 1.8 g/dL, and urine protein to creatinine ratio (UPC) >19.8 mg/mg. Urine microscopy showed 50–100 red blood cells per high power field. Serum complement levels demonstrated mildly decreased level of C3 (66 mg/dL, normal range 82–167 mg/dL) and normal level of C4 (19 mg/dL, normal range 10–34 mg/dL), also noted in Table 1. The patient's hypertension was treated with amlodipine, and he was referred to our institution for further evaluation including consideration of a renal biopsy due to nephrotic syndrome. Based on his clinical and laboratory presentation, our differential diagnosis included IgAVN, immune complex-associated membranoproliferative glomerulonephritis (IC-MPGN), C3 glomerulonephritis (C3GN) in association with genetic or acquired drivers of complement dysregulation, and hypocomplementemic urticarial vasculitis syndrome (HUVS). Diagnosis of HUVS was felt to be less likely given the normal level of C4 typically seen in patients with HUVS (13). Additionally, the rash of HUVS would classically affect the face and upper extremities, whereas this patient's rash was more prominent in the lower extremities. Unfortunately, due to travel distance to our facility and additional psychosocial barriers to care, his evaluation was further delayed by three additional days.

**Table 1:** Serum levels of complement and albumin and urine protein to creatinine ratio (UPC) during the disease course.

**Lab (reference value)**	**Day 18**	**Day 42**	**Day 55**	**Day 70**	**Day 102**	**Day 116**	**Day 130**	**Day 145**	**Day 180**
C3 (82–167 mg/dL)	66	84	—	117	—	—	—	—	—
C4 (10–34 mg/dL)	19	29.5	—	20	—	—	—	—	—
Serum Albumin (3.5–5.2 g/dL)	1.8	1.9	1.5	—	1.9	2.4	2.7	3.0	3.4
UPC (<0.20 mg/mg)	>19.8	>19.8	16.1	8.1	5.3	2.7	4.5	1.7	1.3
Creatinine (mg/dL)	0.54	0.54	0.24	0.21	0.32	0.29	0.28	0.30	0.24

**Figure 1:**
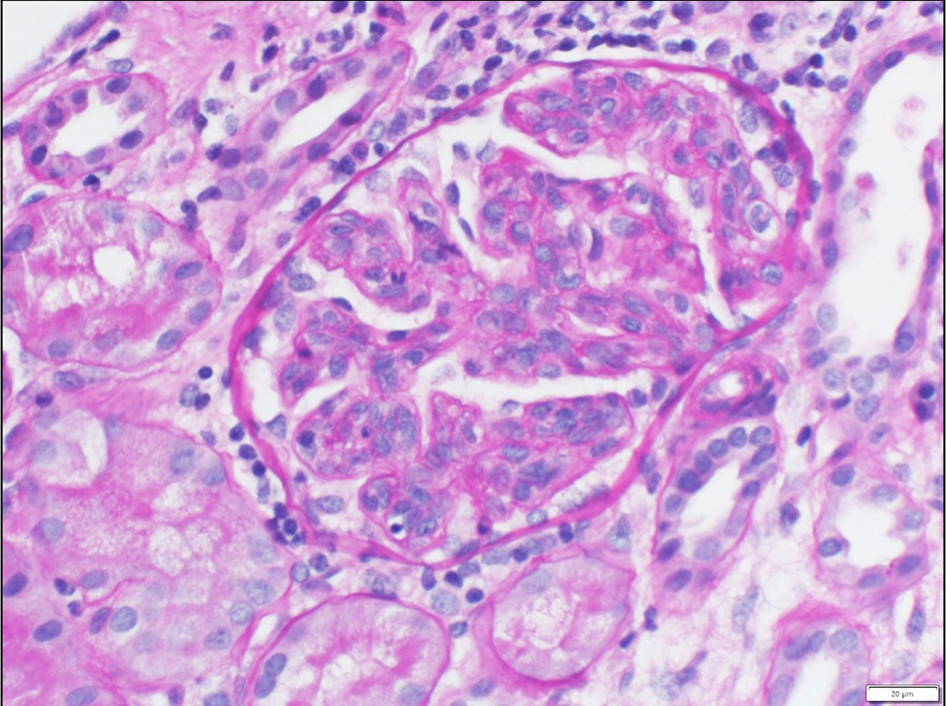
Light microscopy showing prominent lobularization of glomeruli with both mesangial hypercellularity and endocapillary proliferation. Frequent glomerular inflammatory cells are also seen.

**Figure 2:**
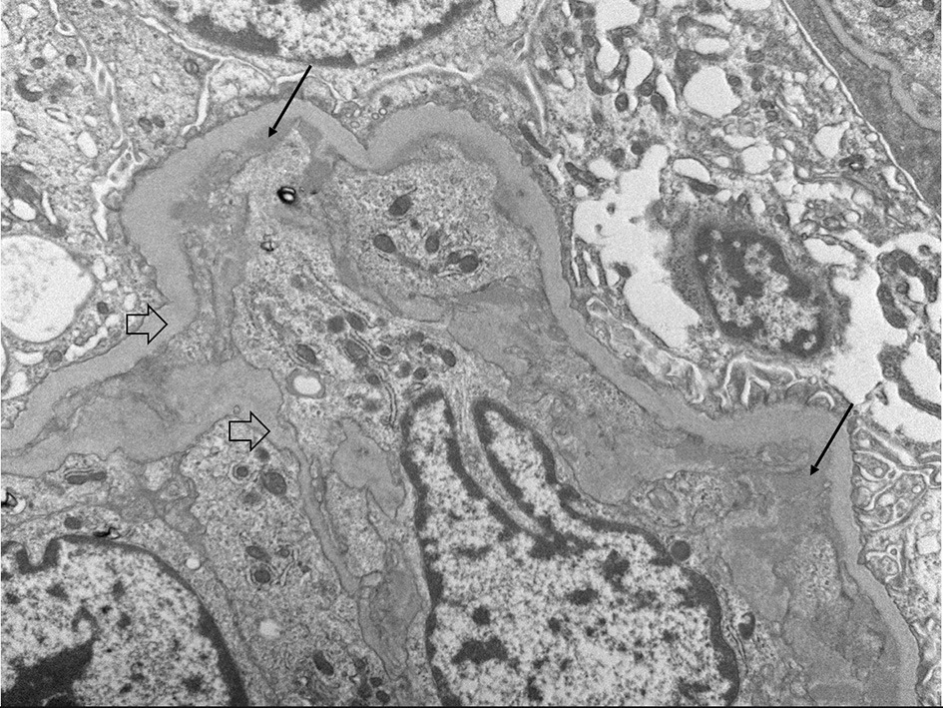
Electron microscopy showing sub endothelial deposits (black arrows) as well as basement membrane duplication or “tram-tracking” (clear arrows).

**Figure 3:**
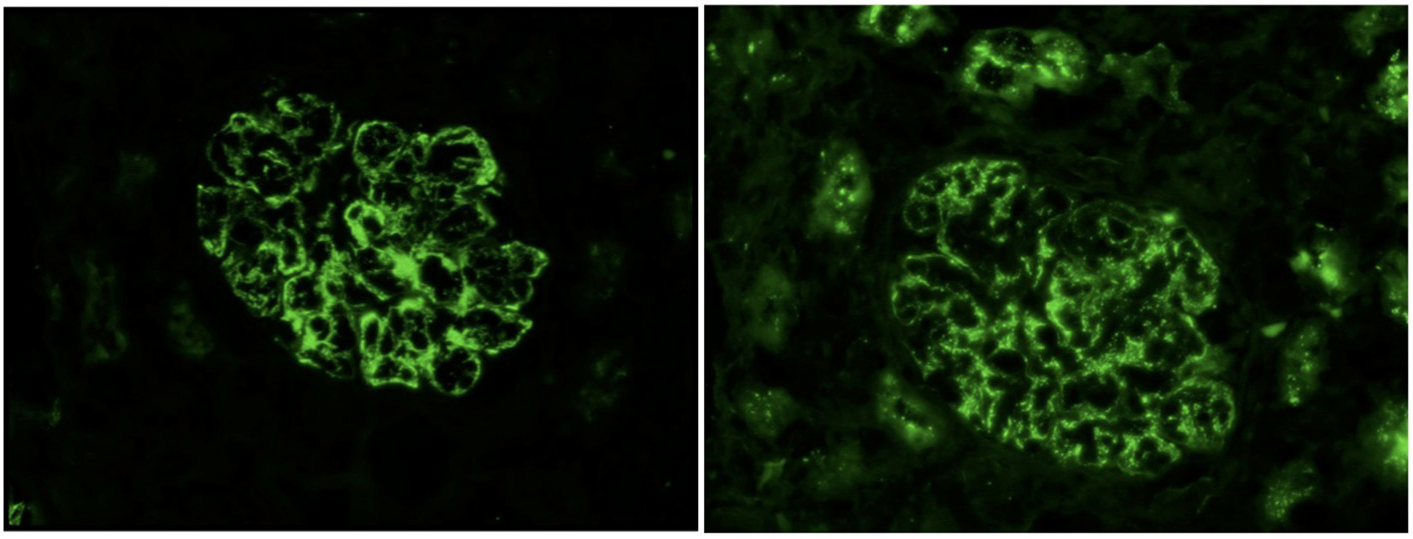
Immunofluorescence showing 2+ IgA deposits **(left)** and 2+ C3 deposits **(right)** with granular capillary loop and mesangial staining.

Upon admission, given the clinical constellation of nephrotic syndrome, the patient underwent percutaneous renal biopsy on Day 22 of his disease course. Light microscopy demonstrated a membranoproliferative pattern of glomerular injury including mesangial and endocapillary hypercellularity, inflammatory cell infiltration, and crescent formation (noted in 3 of 60 glomeruli visualized, see Figure 1). Interstitial fibrosis was absent as noted by Masson trichrome stain, and no tubular atrophy was noted by periodic acid-Schiff stain. Ultrastructural analysis demonstrated electron-dense deposits in the mesangium, paramesangium, and capillary loop basement membrane, with double contour of the glomerular basement membrane (“tram track pattern”) on silver stain (see Figure 2). Immunofluorescence revealed codominant C3 (2+) and IgA (2+) staining in the mesangium and capillary loops, and was negative for IgG, IgM, or C1q staining (see Figure 3). The patient initially received 3 days of pulse IV methylprednisolone. As the biopsy results demonstrated an MPGN pattern of injury with C3 and IgA codominant staining in the capillary loops as well as mesangium, which would be atypical features of IgAV but which could also be consistent with IC-MPGN, the patient's treatment was adjusted on Day 28 of his disease course by the addition of prednisolone 2 mg/kg/day, mycophenolate mofetil (MMF) 450 mg/m^2^/dose twice daily, and lisinopril. In addition, comprehensive quantitative and functional complement testing were performed, which revealed low levels of Factor B, Factor H, and Factor I suggesting activation of the alternative pathway, but with normal levels of Bb, Ba, and properdin which in contrast would be inconsistent with alternative pathway activation. Additionally, the level of C5 was slightly low but sC5b-9 was normal, indicating quiescence of the terminal pathway. No acquired factors of complement dysregulation (C3NeF, C4NeF, C5NeF, Factor H autoantibody, Factor B autoantibody) were noted. Genetic testing has not been pursued to date.

At follow-up, the patient has remained on antihypertensive/antiproteinuric treatment with lisinopril as well as MMF. Mild intermittent swelling persisted but eventually subsided, with his most recent laboratory assessment demonstrate improvements in proteinuria, hypoalbuminemia, and renal function (Table 1). Given this clinical improvement, his prednisone dose is being slowly tapered.

## Discussion

In this patient presenting with purpura, swollen hands, abdominal pain, nephrotic range proteinuria, hypertension, and low serum C3 level, a renal biopsy demonstrated a membranoproliferative pattern of glomerular injury with co-dominant immunofluorescence of C3 and IgA. This histological picture is most consistent with IC-MPGN (14), discordant with the clinical features consistent with IgAVN. However, the timeline of the patient's clinical course appears to fit more with IgAVN, with renal manifestations appearing approximately 2 weeks after the appearance of a purpuric rash (1). IgAVN typically presents with a mesangioproliferative glomerular injury pattern with mesangial hypercellularity and expansion of the mesangial matrix, although it can also be seen in other forms of glomerulonephritis. However, a membranoproliferative pattern is atypical for this entity but has been rarely reported (4, 5). The strong C3 staining with IgA co-dominance and low serum C3 level at presentation suggest dysregulated activation of the complement system as is seen with C3 glomerulopathy (C3G) or IC-MPGN (15), and can act as a disease-modifying factor in IgA nephropathy or IgA vasculitis.

The utility of hypocomplementemia as a prognostic indicator for IgA nephropathy is not clearly demonstrated in the published literature. No correlation between complement and renal outcomes have been demonstrated in pediatric patients (16). However, in a study of Korean adults with IgA nephropathy, low serum C3 levels have been associated with decreased renal survival (17). Studies examining the correlation of the intensity of mesangial C3 staining with renal survival have had inconsistent findings (17, 18). Based on these results, the prognostic indicators need further study but is promising.

In our patient, the hypocomplementemia, MPGN pattern of glomerular injury, and co-dominant C3 and IgA immunofluorescence on renal biopsy suggested a more pronounced role of complement in the pathogenesis of his IgAVN. This has led to treating the patient with a regimen of immunosuppression typical in the treatment of patients with IC-MPGN and C3G (19–22), with a concomitant improvement in the patient's hypocomplementemia, proteinuria, hypoalbuminemia, and renal function after 3 months of aggressive treatment. Looking through the literature, one case report presented a patient similar to ours who recovered renal function after 2 years with corticosteroid monotherapy; however, this patient did not have a comprehensive complement evaluation (23).

The description of this case has several limitations. One such limitation includes a lack of availability of skin biopsy to confirm a diagnosis of IgAV due to delay in the patient presenting to our institution. Additionally, genetic testing for variants in complement regulatory genes was deferred out of concern for undue financial burden to the family arising from this testing. Although a co-existent acquired factor causing complement dysregulation (i.e., C3NeF) was not identified in this patient, pathogenic variants in complement regulatory genes or a risk haplotype as seen in C3G and IC-MPGN may still be present and warrant exploration (18). The speculated role of dysregulated complement activation in this patient's renal lesion and clinical course may suggest the utility of investigating complement-targeted therapy as has been reported in cases of refractory IgA nephropathy (24). Ultimately, such patients may benefit from the panoply of complement-targeted therapeutic agents that are currently in clinical development (24, 25).

## Patient Perspective

After discharge from the hospital, my son had to see his pediatrician weekly. For four months, we struggled with swelling of his eyes, leg, and scrotum and his blood pressure. His symptoms eventually improved, and we were able to go down on his medications. Now, he is off of his steroids and is only on one blood pressure medication.

## Data Availability Statement

The original contributions presented in the study are included in the article/supplementary material, further inquiries can be directed to the corresponding author.

## Ethics Statement

Ethical review and approval was not required for the study on human participants in accordance with the local legislation and institutional requirements. Written informed consent to participate in this study was provided by the participants' legal guardian/next of kin. Written informed consent was obtained from the individual(s), and minor(s)' legal guardian/next of kin, for the publication of any potentially identifiable images or data included in this article.

## Author Contributions

All authors listed have made a substantial, direct, and intellectual contribution to the work and approved it for publication.

## Conflict of Interest

The authors declare that the research was conducted in the absence of any commercial or financial relationships that could be construed as a potential conflict of interest.

## Publisher's Note

All claims expressed in this article are solely those of the authors and do not necessarily represent those of their affiliated organizations, or those of the publisher, the editors and the reviewers. Any product that may be evaluated in this article, or claim that may be made by its manufacturer, is not guaranteed or endorsed by the publisher.
